# Atrial Cardiomyopathy: An Unexplored Limb of Virchow's Triad for AF Stroke Prophylaxis

**DOI:** 10.3389/fcvm.2020.00011

**Published:** 2020-02-18

**Authors:** Ashley Darlington, Mark D. McCauley

**Affiliations:** ^1^Division of Cardiology, Department of Medicine, College of Medicine, University of Illinois at Chicago, Chicago, IL, United States; ^2^Jesse Brown VA Medical Center, Chicago, IL, United States; ^3^Department of Physiology and Biophysics, University of Illinois at Chicago, Chicago, IL, United States

**Keywords:** atrial cardiomyopathy, atrial fibrillation, atrial contractility, stroke, Virchow's triad

## Abstract

The most dreaded complication of atrial fibrillation is stroke, and 70–80% of patients with AF-related stroke die or become disabled. The mechanisms of thromboembolism in AF are multifactorial, with evidence demonstrating that all three criteria of Virchow's triad are satisfied in AF: abnormal stasis of blood, endothelial damage, and hypercoagulability. Mechanistic insights into the latter two limbs have resulted in effective stroke prophylactic therapies (left atrial appendage occlusion and oral anticoagulants); however, despite these advances, there remains an excess of stroke in the AF population that may be due, in part, to a lack of mechanistic understanding of atrial hypocontractility resulting in abnormal stasis of blood within the atrium. These observations support the emerging concept of atrial cardiomyopathy as a cause of stroke. In this Review, we evaluate molecular, translational, and clinical evidence for atrial cardiomyopathy as a cause for stroke from AF, and present a rationale for further investigation of this largely unaddressed limb of Virchow's triad in AF.

## Introduction

The atria play an integral role in the initiation of the cardiac cycle, maintenance of sinus rhythm, ventricular filling, and cardiac output (1). First, the atria house both the sinoatrial and atrioventricular nodes, which are responsible for initiation of normal cardiac rhythm and its transduction to the ventricles, respectively. Second, the atria are a reservoir for venous return during systole, and a conduit and booster pump for ventricular filling during diastole. In addition to its unique function in the cardiac cycle, the atria have specialized structural and electrophysiologic characteristics that are distinct from ventricular myocytes and which include: the expression of connexin-40, the presence of I_Kur_ and I_KAch_ channels, smaller I_K1_ current and less negative resting cell membrane potential, and expression of unique myofilament proteins (such as atrial myosin light chain 2, MLC2a). Yet, despite the significant differences in structure, function, and molecular/cellular content between atria and ventricles, there is comparatively much less known about atrial cardiomyopathy (AC) than is known about ventricular heart failure and cardiomyopathy.

This gap in knowledge is clinically significant in patients with a high risk of atrial fibrillation (AF) and stroke. AF, the most common sustained arrhythmia, increases stroke risk five-fold and is an independent risk factor for thromboembolism, heart failure and impaired quality of life (2). Stroke remains the most dreaded and deadly complication of AF, as 70–80% of patients with AF-related stroke die or become disabled (3). The mechanisms of thrombogenesis related to AF are multifactorial, and there is evidence to suggest that all three criteria of Virchow's triad are satisfied in AF: abnormal stasis of blood, endothelial damage, and hypercoagulability (4). Specific pro-thrombotic conditions in AF include atrial dilation, atrial hypocontractility, endothelial hypertrophy and fibrosis, and secretion of pro-thrombotic factors including IL-6 and von Willebrand factor (5, 6). Current stroke prophylactic therapies in AF include oral anticoagulants and left atrial appendage (LAA) exclusion, which address two of the three factors of Virchow's triad, namely hypercoagulability of blood and exclusion of the most thrombogenic portion of the left atrium, the LAA (7). However, both therapies have significant side effects and risks that limit their use. Direct oral anticoagulants (DOACs) increase bleeding risk, have significant drug-drug interactions, are limited by renal function, and are difficult to reverse. Warfarin has a narrow therapeutic window, high hemorrhage rate, and monitoring/compliance issues (8). Likewise, LAA exclusion has significant intraoperative risk, and recent reports have shown a thrombus formation rate of 3.7% (9, 10).

Importantly, there is no prophylactic agent against stroke that addresses atrial hypocontractility as a primary mechanism. This is likely due to lack of mechanistic understanding of how atrial contractility is regulated in AF. Given the importance of atrial hypocontractility in thromboembolism formation, elucidation of the mechanisms underlying atrial hypocontractility in AF remains a major research priority.

Atrial hypocontractility is well-described on echocardiography where spontaneously coagulating blood fills the left atrium during AF (11). Detailed echocardiographic measurements of atrial wall velocities in human AF patients show that there is a significant reduction of atrial contractility (12, 13). Likewise, atrial hypocontractility and atrial dilation are associated with increased risk of stroke (1). Several clinical observations suggest that in addition to electrical ion channel remodeling in AF, atrial contractile dysfunction forms a substrate for stroke risk: (1) atrial hypocontractility persists for 6–8 weeks following conversion from AF to sinus rhythm (14, 15); (2) atrial dilation and hypocontractility may progress despite catheter ablation of AF, leading to stroke (16, 17); (3) there is a temporal dissociation between the occurrence of AF and the development of stroke (18); and (4) atrial hypocontractility alone is an independent risk factor for stroke (19, 20). These observations support the emerging concept of AC as a cause of stroke, and which may have both mechanical (contractile) and electrical (AF) manifestations. Recently, to address these observations, a consensus document was published to establish the definition and clinical implications of AC; AC is defined as: “any complex of structural, architectural, contractile, or electrophysiological changes affecting the atria with the potential to produce clinically-relevant manifestations” (1). Thus, the concept of AC as a contributor to cardiovascular disease, and as a separate but related entity from ventricular cardiomyopathy, has gained momentum as of late. This review article discusses AC and the investigations underlying our increasing understanding of the atrial myopathic substrate as a risk factor for thromboembolism and stroke.

## Initial Observations Suggesting the Atrial Cardiomyopathy Phenotype

In 1972, Nagle et al. were the first to describe the term “atrial cardiomyopathy” in reference to a family in which three of five siblings demonstrated first degree heart block and ectopic supraventricular tachycardia, progressing to persistent atrial standstill with complete loss of response to direct atrial stimulation (21). Over a decade later, Stables et al. described a similar family cohort with reduced atrial contractility, intermittent atrial standstill, diffuse atrioventricular block, and a systemic embolism (22). This observation was important because for the first time it linked atrial contractile dysfunction with thromboembolism formation. In 1997, Jaïs et al. described nine patients with paroxysmal AF who were found to have a focal source primarily in/near the pulmonary veins (23); these findings and others led Zipes et al. to recognize that AF and other supraventricular tachycardias cause AC and vice versa (24, 25). Further work by Hoit et al. and Sun et al. demonstrated that atrial tachypacing in dogs is associated with atrial enlargement, atrial contractile dysfunction, and reduced Ca^2+^ transients from remodeling of intracellular Ca^2+^ handling proteins such as phospholamban (26, 27). These seminal observations set the stage for subsequent genetic, cellular, and translational investigations into the mechanisms of atrial cardiomyopathy.

## Genetic Inputs into the Atrial Cardiomyopathy Phenotype

As the consensus definition of AC is broad, it is useful to sub-divide genetic determinants of AC into primary and secondary causes. Primary genetic AC refers to atrial genes that are functionally active in the atria and which contribute either atrial development and/or the maintenance of atrial structural, electrical, and metabolic properties (28). *MYL4*, the gene that encodes a fetal isoform of atrial myosin light chain 2 (MLC2a), is an example of a structural myofilament gene that is uniquely expressed in the atria, and in which genetic mutations can manifest in atrial contractile dysfunction and AF. Peng et al. described a family with a rare missense loss-of-function *MYL4* variant resulting in a phenotype demonstrating progressive atrial-selective electromechanical dysfunction, tachyarrhythmias, and bradyarrhythmias requiring pacemaker implantation (29). In this study, the group also created a knock-in rat model to demonstrate the mutation causes proapoptotic and profibrotic signaling, along with increased atrial cardiomyocyte terminal deoxynucleotidyl transferase dUTP nick end labeling staining, suggesting enhanced apoptotic cell death. More recently, Zhong et al. described a single-nucleotide polymorphism (rs4968309) in *MYL4* which is associated with atrial dilation, increased AF, and AF recurrence after cryoballoon AF ablation (30). Another example of a primary genetic input to AC is the *NPPA* gene, which encodes for atrial natriuretic peptide (28). Disertori et al. described *NPPA* variants in three families from Northern Italy who developed giant biatrial dilatation, supraventricular arrhythmias, atrial standstill, and cardiac thromboembolism (31).

Secondary genetic AC refers to atrial structural and electromechanical dysfunction in gene mutations known to contribute to other cardiovascular diseases such as: Brugada Syndrome, genetic AF syndromes (*PITX2* gene), and genetic muscular dystrophies. Table 1 lists the genes, mutations, phenotypes, and references for relevant primary and secondary inputs to genetic AC. Although these insights have been important for elucidating the genetic inputs into AC and subsequent AF, the majority of patients with AF have significant acquired risk factors affecting atrial cellular function and which are described below.

**Table 1:** Primary and secondary genetic inputs into atrial cardiomyopathy.

**Gene**	**Mutation**	**Phenotype**	**References**
**PRIMARY GENETIC INPUTS**			
*MYL4*	p. (E11K)	Atrial tachyarrhythmias, atrial standstill, severe bradyarrhythmias	(29)
*MYL4*	SNP (rs4968309)	Atrial dilation, increased AF, AF recurrence after cryoballoon PVI ablation	(30)
*NPPA*	p. (R150G)	Giant biatrial dilatation, supraventricular arrhythmias, atrial standstill, cardiac thromboembolism	(31)
**SECONDARY GENETIC INPUTS**			
*SCN5A*	Multiple	Brugada Syndrome, giant biatrial dilatation, early arrhythmias with progression to atrial standstill, thromboembolic complications	(32)
*PITX2*	M207V	Early onset AF, atrial dilation	(33)
*DMD*	Multiple	Duchenne and Becker muscular dystrophies	(1)
*DMPK*	Multiple	Type 1 muscular dystrophy	(1)
*LMNA*	Multiple	Lamin A/C cardiomyopathy with conduction system disease	(1)

## AC in the Basic Sciences and Cell Metabolism

Recent basic science studies into the cellular mechanisms of AC have provided significant insights into atrial Ca^2+^ handling, contractility, metabolism, and apoptosis. First, disorders of Ca^2+^ handling, resulting in intracellular Ca^2+^ overload, are associated with reduced atrial contractility and increased atrial fibrosis. Endothelin-1 (ET-1) is a vasoconstrictor peptide and a biomarker of endothelial damage, cardiac fibrosis, and decreased atrial contractility. Increased ET-1 in atrial fibrillation is associated with atrial pre-excitation, inappropriate Ca^2+^ leak, and intracellular Ca^2+^ overload associated with left atrial (LA) dilation and fibrosis (34). Liver kinase B1 (LKB1), is highly expressed in the heart, and is responsible for cell signaling responses regulating myofilament response to Ca^2+^, or Ca^2+^ sensitivity. Ozcan et al. demonstrated that mice with a cardiac-specific knockout of *Lkb1* developed early-onset atrial myocarditis, associated with severe bi-atrial enlargement, atrial thrombus, and early spontaneous AF (35). μ-calpain, an intracellular Ca^2+^-activated protease which mediates the actions of calcium, was shown to be elevated in 16 patients with paroxysmal AF vs. sinus rhythm, and leads to the destruction of contractile filaments in fibrillating atria, thereby enhancing atrial remodeling and reducing atrial contractility (36, 37). Second, primary electrical (ion channel) remodeling of the atria has been associated with AC. The two-pore-domain potassium channel TASK-1 helps regulate atrial action potential duration. Wiedmann et al. demonstrated that decreased TASK-1 expression in AF-prone Crem-IbΔC-X transgenic mice is associated with both AC and AF (38, 39). Likewise, mutations in the voltage-gated sodium channel have been reported to be associated with severe left atrial dilation and atrial standstill (40). Third, progressive atrial contractile dysfunction has been demonstrated in diseases affecting cardiac metabolism. Obesity and diabetes are common diseases involving myocardial inflammation and expansion of epicardial adipose tissue; several groups have described a strong correlation between epicardial adipose tissue and atrial myopathy leading to atrial dilation and AF (41–44). In particular, diabetes has been associated with significantly reduced global atrial strain rate and LA volume, independently of AF status (45). Additionally, mitochondrial dysfunction resulting from atrial oxidation in AF has been associated with mitochondrial DNA damage and contributes to a “vicious” cycle of oxidative stress and progressive atrial myopathy in AF (46). These studies firmly link AC to disorders of Ca^2+^ handling, both in the release and reuptake of intracellular Ca^2+^ and the myofilament response to cytosolic (Ca^2+^), and present a rationale for future study of molecular targets that may preserve Ca^2+^ homeostasis in diseases that introduce metabolic or oxidative stress.

However, beyond these strictly myocardial inputs, the AC substrate also favors AF development from the progressive development of atrial fibrosis, which serves as a nidus for focal electrical slowing within the atria and as an anchor point for micro-reentrant circuits within the atria.

## Fibrosis and Structural Atrial Remodeling

Closely related to atrial contractile dysfunction in AC is the occurrence of atrial fibrosis, which is an integral part of atrial remodeling in AF. The majority of patients with AF and without known structural heart disease have atrial fibrosis as a substrate (47). Fibrosis is also a prominent histopathologic and mechanistic feature of early persistent AF (48). Despite these insights, the burden of atrial fibrosis does not always directly correlate to AF burden; patients with very low atrial fibrosis may have persistent AF, while patients with higher fibrosis may or may not manifest in higher AF burden (48). Clearly, atrial fibrosis is just one piece of the complex mechanistic puzzle of AF.

Several causes for enhanced atrial fibrosis have been described. Aging is the most common risk factor for atrial fibrosis. Given that aging is associated with oxidative stress, Ca^2+^ dysregulation, and apoptosis, it is not surprising that age is directly associated with the extent of both atrial fibrosis and apopotosis (49). Matrix metalloproteins are highly expressed in AF and positively correlate to extent of atrial fibrosis in AF; in particular matrix metalloprotein 9 (MMP-9) has been described as a circulating serum biomarker suggestive of atrial fibrosis (47). Additionally, alcohol consumption is associated with progressive fibrosis of the atria. McManus et al. analyzed data from the Framingham Heart Study and determined that for every 10 g of alcohol consumed daily, a 0.16 mm enlargement of the left atrium occurred with progressive fibrosis (50). Thus, atrial fibrosis is highly correlated with AC and is a marker of progressive atrial dilation, hypocontractility, and AF susceptibility. The combination of these risk factors contributes to the complex interplay of atrial movement and inflammation and hypercoagulable milieu at the atrial endocardial border.

## Hypercoagulability May Contribute to the AC Phenotype

Multiple studies have shown increased thrombogenesis and platelet activation in patients with atrial dysfunction, as is seen in AC (51, 52). Tsai et al. demonstrated that fibrinopeptide A, a prothrombotic marker, is associated with marked left atrial appendage (LAA) spontaneous echo contrast (SEC), a marker of atrial contractile dysfunction (53). Sakurai et al. later showed that among patients with atrial flutter, those with LAA SEC were associated with increased serum D-dimer and reduced B-thromboglobulin, biomarkers of platelet activation (54). Igarashi et al. confirmed this association in patients with atrial fibrillation, demonstrating that LAA SEC was associated with increased D-dimer and thrombin-antithrombin III complex (55).

The hypercoagulable state found in AF contributes to stroke risk through two primary mechanisms: (1) increased clot formation which is an independent limb of Virchow's Triad, and (2) through progressive atrial remodeling that drives formation of the AC substrate. Recently, Spronk et al. demonstrated that in isolated rat atrial fibroblasts, thrombin enhances the phosphorylation of pro-fibrotic signaling molecules Akt and Erk, and increases expression of transforming growth factor β1 (TGF-β1) and the pro-inflammatory monocyte chemoattractant protein-1; all of these effects were attenuated by the direct thrombin inhibitor dabigatran (56). Furthermore, in six goats with persistent AF, treatment with nadroparin, which targets Factor Xa-mediated thrombin generation, reduces the complexity of the AF substrate as measured by LA maximal activation time differences and quantification of endomysial fibrosis (56). Similarly, Hasan et al. showed in porcine endothelial cells, thrombin induced death and contributed to adverse angiotensin II signaling (57). Treatment with thrombin resulted in increased oxidative stress via NADPH oxidase, cyclooxygenases and the mitochondrial respiration complex, as well as enhanced expression of: vascular cell adhesion molecule 1 (VCAM-1), TGF-β, and matrix metallopepditases 2 and 9 (MMP-2 and-9). This treatment also resulted in overexpression of angiotensin converting enzyme and angiotensin-1 receptors, a signaling pathway that is known to contribute to remodeling of the myocardium (57). Importantly, this adverse angiotensin II signaling was reversed when treated with angiotensin converting enzyme inhibitors (ACEI) or angiotensin receptor blockers (ARB) (57). Combined, these results suggest that inhibition of coagulation may reduce stroke risk not only by reducing thromboembolism, but also by limiting progression of the AC substrate for AF. Conversely, inhibition of the renin-angiotensin pathway with ACEI may reduce the pro-coagulant milieu at the atrial endothelial border.

## Atrial Imaging and Prognostic Implications

Atrial fibrosis is a hallmark imaging finding seen in structural remodeling that results in AC. Fibrosis can be measured non-invasively by delayed gadolinium enhancement on cardiac magnetic resonance imaging (cMRI). The burden of fibrosis is evaluated as a percentage of total LA wall volume and then categorized into Utah stages I (<10% fibrosis), II (10–20% fibrosis), III (20–30% fibrosis), and IV (>30% fibrosis) (58). Measurement of atrial fibrosis can assess mechanical function, as greater fibrosis is associated with LAA dysfunction (59) and reduced atrial systolic function (60). Importantly, its measurement can also provide insight into the predicted clinical course, as greater atrial fibrosis is also associated with stroke (61) and arrhythmia recurrence post-ablation (62).

Atrial strain by speckle-tracking echocardiography provides similar functional and clinical information through a more readily available imaging modality. Atrial strain measurements directly reflect the structural and electrical remodeling driving the development of atrial cardiomyopathy. Decreased atrial strain is inversely correlated with atrial fibrosis (63) and prolonged conduction time (64). Furthermore, atrial strain can predict clinical course and response to treatment modalities. Reduced atrial strain is associated with LAA dysfunction, LAA thrombus, and stroke independent of the presence of AF (65–67). Patients with reduced atrial strain are more likely to have poor response to treatments including unsuccessful electrical cardioversion (65), and arrhythmia recurrence after ablation (68). Interestingly, reduced atrial strain is also associated with increased mortality after stroke, and new onset AF after stroke, supporting AC as a primary driver of stroke risk independent of arrhythmic burden (20, 69).

## AC and Dementia

The relationship between AF and dementia has long been established (70). However, recent insights have demonstrated that there may also be an association between AC and dementia (71). For example, in a subgroup analysis of the Atherosclerosis Risk in Communities Study (ARIC) O'Neal et al. demonstrated that advanced intra-atrial block on ECG, a strong indicator of left atrial remodeling present in AC, is associated with increased stroke risk and dementia risk (72). However, AF has also been independently correlated with increased risk for cognitive decline and dementia (hazard ratio 1.23) independent of cardiovascular risk factors including ischemic stroke (73).

One proposed mechanism for this association is non-uniform cerebral blood flow in patients in persistent AF. Physiologic studies of cerebral perfusion demonstrate the variable cardiac output produced during AF results in critical hypoperfusion at the arteriolar level and excessive hypertension at the capillary level (74). Mouse models have demonstrated that chronic cerebral hypoperfusion is associated with decline in learning and memory. For example, compared with controls, mice with bilateral occlusion of common carotid arteries had greater amyloid-beta deposition in the hippocampus (75). This study also identified higher concentrations of soluble amyloid precursor proteins and their myloidogenic processing proteins. This could be a possible mechanism explaining the association between AF-related cerebral hypoperfusion and Alzheimer's Disease. This data suggests there may be an indirect link between AF-related cerebral hypoperfusion and Alzheimer's Disease.

Dementia risk may be reversible when sinus rhythm can be maintained using AF ablation. Bunch et al. demonstrated that in patients that underwent catheter ablation of AF, incidence of dementia at 3 years was reduced to the level of patients without AF (occurrence rate: 1.9% AF without ablation, 0.4% AF with ablation, 0.7% no AF; *p* < 0.0001) (76). These data suggest that early intervention into AF may alter the AC substrate and may collectively reduce long-term risk of dementia as well as stroke risk. Further prospective, randomized controlled trials are necessary to validate these results and provide a stronger indication for early AF substrate modification.

## AC in Electrophysiology Studies and EP Interventions

The concept of AC has been translated not only to cardiovascular imaging, but also to inform decision-making in interventional electrophysiology procedures. First, regarding the substrate for AF ablation, substrate progression is now seen as a multifactorial response of cardiomyocytes to electrical, mechanical, and metabolic stressors (77). While some components of these changes to the LA are reversible (or adaptive), others such as progressive fibrosis are more permanent, or maladaptive (77). Currently, there is a lack of standard techniques to pre-operatively assess the degree of atrial fibrosis and to identify the potential rotors for AF, which could theoretically improve the results of catheter ablation, especially in patients with long-standing and persistent AF (78). Regarding mapping and AC, there has been significant progress in developing multi-modality inputs into standard electroanatomic mapping techniques to achieve a greater understanding of arrhythmic substrate. One example of a multi-modality approach includes the focal impulse and rotor (FIRM) mapping technique, which takes advantage of re-entrant driver “rotors” which are mostly localized in the patchy zones bordering areas of dense fibrosis seen on atrial MRI (79). MRI coupled with electroanatomic mapping has been useful for defining the arrhythmogenic substrate in patients with rare genetic causes of familial AF, such as a recently described family with a mutation in *NPPA*, as discussed above (31, 80).

For ablation of supraventricular arrhythmias, focal (fibrotic) AC has been a challenge. Women appear to show fibrotic AC at a higher rate than men as recently reported in a Swiss study of box isolation of fibrotic areas (BIFA) for AF (81). Although the cause for this higher rate is incompletely known, older age and circulating galactin III biomarkers in the female population (ratio: 1 female: 1.4 male) undergoing the procedure was postulated to contribute to this observation (81, 82). While the distribution of LA fibrosis is variable among AF patients, the anterior LA is especially pronounced and vulnerable to both fibrosis and focal drivers of AF. In patients with complex atrial anatomy, such as patients with prior valve surgery, history of atrial ablation procedures, and fibrotic AC, complex ablation lesions may result in lateral RA isolation or even complete sinus exit block with bradycardia requiring pacemaker implantation (83). Regarding recurrence of arrhythmias, atrial fibrosis is an independent risk factor for AF recurrence after pulmonary vein isolation without LA substrate modification (84). Employment of substrate modification techniques or repeat ablations paradoxically increase the burden of fibrosis. Under the new perspective of AC as a driver for AF, increasing atrial fibrosis through ablation techniques could expedite disease progression.

There are no reliable serum biomarkers that predict both the burden of atrial fibrosis and also the recurrence of AF after ablation (78). Thus, insights into the AC substrate with cardiac imaging and catheter-based approaches allow for optimal prognostication and treatment of AF and other supraventricular arrhythmias.

## Future Directions: From Virchow's Triad to New Treatments for AC

**Figure 1:**
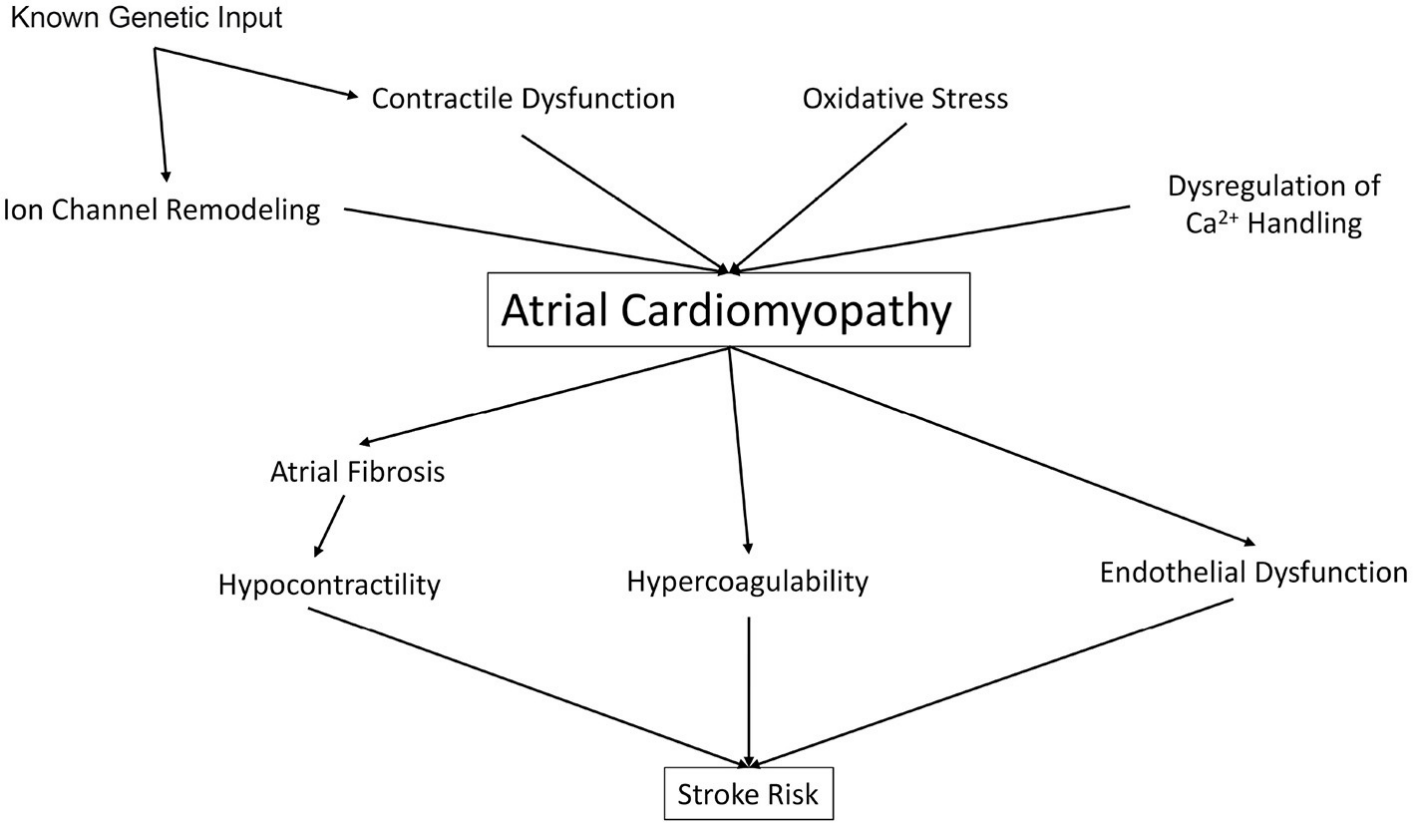
Atrial cardiomyopathy risk factors increase risk for stroke.

Whereas, Virchow's Triad has set the stage for the understanding of vascular thrombosis, and has implicated AC as a significant driver of thromboembolic stroke, the insights discussed above (Figure 1) present a rationale for an updated model of stroke which includes both intracardiac factors (such as atrial and ventricular contractility) and extracardiac factors (including traditional CHADS-VASc and liver/coagulopathy) as well. First, despite the link between AF and stroke, maintenance of sinus rhythm with anti-arrhythmic drugs (AAD) has not been shown to reduce stroke risk and overall CV mortality vs. rate control therapy (1). Second, many AAD (such as Vaughn-Williams Class I AAD flecainide, Class III Sotalol, and Class IV diltiazem) are contraindicated in patients with reduced cardiac contractility and heart failure. Catheter ablation of AF often results in impairment of LA systolic function, with both a decrease in atrial ejection fraction, and also a lack of meaningful “a” waves on pressure tracings, suggesting prolonged impairment of atrial mechanical function (85). Furthermore, catheter ablation may “influence ongoing pathologies” such as increased atrial size, structure, and mechanical atrial function, and thus affect future risk for atrial thrombogenesis (1). Given these limitations, efforts are underway to address reduced atrial contractility in AF.

For example, dysregulation of the myofilament response to Ca^2+^ release, or Ca^2+^ sensitivity, is a significant contributor to contractile dysfunction in AF (51, 86). Contractility and intracellular Ca^2+^ homeostasis rely upon phosphorylation processes, which in AF are pathologically altered (87). In patients with chronic AF, there is an increase in total protein phosphatase 1 (PP1) activity, which is associated with dephosphorylation of key regulatory proteins involved in Ca^2+^ homeostasis and Ca^2+^ sensitivity (86, 88). Additionally, experimental evidence supports the role of PP1 subunit expression in the dysregulation of Ca^2+^ handling in AF. Chiang et al. performed a detailed PP1 interactome analysis in human paroxysmal AF, and determined that alterations in PP1 regulatory subunit (PP1R) and catalytic subunit (PP1c) binding underlie pathologic signaling for several atrial Ca^2+^ handling proteins (89). Of these interactions, only one regulatory subunit—protein phosphatase 1 regulatory subunit 12C (PPP1R12C)—targets the sarcomere at a key regulatory site for myofilament Ca^2+^ sensitivity: Atrial myosin light chain 2 (MLC2a). Previously, Perike et al. have shown that human AF patients experience a 3.5-fold increase in PPP1R12C protein expression in AF. In a genetic mouse model overexpressing the PPP1R12C protein to similar levels, we observed a 150% increase in atrial diameter, reduced atrial strain, and an increase in AF susceptibility, ultimately contributing to the AC substrate (90). Future studies are needed to determine whether reduction in PPP1R12C expression or inhibitors of PPP1R12C protein activity are sufficient to suppress the AC substrate in AF and ultimately prevent stroke.

Another therapeutic target of interest in improving atrial contractility is the Ca^2+^- sensitizing agent levosimendan. Initially, levosimendan was shown to increase AF in both the second Randomized Multicenter Evaluation of Intravenous Levosimendan Efficacy (REVIVE II) and the Survival Of Patients with Acute Heart Failure in Need of Intravenous Inotropic Support (SURVIVE) studies (91, 92). However, more recent studies have shown that levosimendan increases cerebral blood flow, decreases NT-pro-BNP (a contributor to adverse atrial remodeling), improves atrial booster function on echocardiography (93). Furthermore, experimental observations in rabbits suggest that predisposition to AF in levosimendan-infused hearts may be attenuated (and nearly abolished) by concomitant treatment with ranolazine, an inhibitor of the late inward sodium current (94). These new data offer an opportunity to “revive” the potential use of levosimendan for both atrial and ventricular contractility, with the help of an AF-limiting agent, ranolazine; further clinical studies will be needed to confirm the efficacy of this approach in human patients.

In addition to approaches that increase atrial contractility, there have been efforts to use existing cardiovascular drugs to preserve remaining atrial myocardial function and limit the progression of atrial fibrosis. The most notable example has been in the area of renin-angiotensin system (RAS) inhibition. Pathologic secretion of angiotensin II occurs both in AF and heart failure (HF) and contributes to atrial structural remodeling and promotes atrial fibrosis, both of which cause AC and AF (95). Experimental evidence in dogs shows that treatment with ACEI drug enalapril reduces tachypacing-induced atrial remodeling, *in vivo* (96, 97). *Post-hoc* analyses from several randomized controlled clinical trials have shown that ACEI and ARB may have a role in preventing new-onset AF (98–100); a recent meta-analysis of nine relevant clinical trials showed that ACEI and ARB had an 18% overall effect in risk reduction for new-onset AF, although this effect was most pronounced (43%) in patients with heart failure (101). Following this analysis, a prospective randomized placebo-controlled multicenter trial with ARB drug olmesartan (ANTIPAF) did not show that 1 year of ARB therapy could reduce the number of AF episodes in patients without structural heart disease (102). Additionally, in a Swedish cross-sectional study of post-hospitalization outcomes from HF and/or AF, RAS inhibition with ACEI or ARBs was associated with a lower risk of all-cause mortality, but was not associated with a lower incidence of new-onset AF (103). These results show that treatment of hypertension, a modifiable risk factor for AF, may affect the AC substrate, and suggest that partial modification of the AC substrate is possible in hypertensive patients at risk for AF.

Finally, recent efforts have also focused on the clinical interaction between obesity and AC contributing to AF. An experimental model of sheep being fed a high-calorie diet showed left atrial enlargement, increase in inflammatory biomarkers, reduction of AERP, and an increase in AF susceptibility (104). In human patients, body mass index (BMI) is an independent risk factor for both atrial remodeling and AF risk (105). Additionally, reduction of BMI is shown to be protective both to AC and AF risk. The Swedish Obese Subjects (SOS) study demonstrated that a 20% weight reduction with bariatric surgery significantly reduced AF risk vs. those with traditional non-surgical treatment (106). Importantly, the distribution of fat may also play an important role in AF risk. Several observational studies have shown that epicardial fat volume, but not pericardial or intra-abdominal fat volume, was independently associated with AF in a dose-dependent manner (107–109). Through direct contact with the myocardium, it is postulated that the epicardial fat pad could contribute to development of the AC substrate through two mechanisms: (1) direct infiltration causing alteration in electrical properties and (2) providing a reservoir for inflammatory and oxidative modulators (110). Future prospective trials will be necessary to determine BMI and weight distribution thresholds necessary for optimal AF prevention.

## Conclusions

Virchow's triad is a useful basis for the understanding of stroke risk in AF. While oral anticoagulants and LA appendage exclusion address two limbs of Virchow's triad, atrial hypocontractility and stasis of blood is a significant unaddressed limb that result in excess stroke risk. AC is now recognized as a significant contributor to atrial hypocontractility and a substrate for AF in susceptible populations. Coupled with traditional CHADS-VASc stroke risk factors, AC is becoming a new potential target for both drug and catheter based treatments. The real challenge moving forward is to translate recent basic science and clinical findings into a mechanism-based therapy to suppress AC and atrial stunning as a contributor to atrial remodeling in AF.

## Author Contributions

AD and MM were responsible for writing, editing, and submitting the above work.

### Conflict of Interest

The authors declare that the research was conducted in the absence of any commercial or financial relationships that could be construed as a potential conflict of interest.

## References

[1] Goette A Kalman JM Aguinaga L Akar J Cabrera JA Chen SA. EHRA/HRS/APHRS/SOLAECE expert consensus on atrial cardiomyopathies: Definition, characterization, and clinical implication. Heart Rhythm. (2017) 14:e3–40. 10.1016/j.hrthm.2016.05.028 27320515 PMC5548137

[2] Mou L Norby FL Chen LY O'Neal WT Lewis TT Loehr LR. Lifetime risk of atrial fibrillation by race and socioeconomic status: ARIC study (atherosclerosis risk in communities). Circ Arrhythm Electrophysiol. (2018) 11:e006350. 10.1161/CIRCEP.118.006350 30002066 PMC6053683

[3] Gladstone DJ Spring M Dorian P Panzov V Thorpe KE Hall J. Atrial fibrillation in patients with cryptogenic stroke. N Engl J Med. (2014) 370:2467–77. 10.1056/NEJMoa1311376 24963566

[4] Watson T Shantsila E Lip GY. Mechanisms of thrombogenesis in atrial fibrillation: Virchow's triad revisited. Lancet. (2009) 373:155–66. 10.1016/S0140-6736(09)60040-4 19135613

[5] Eckstein J Verheule S de Groot NM Allessie M Schotten U. Mechanisms of perpetuation of atrial fibrillation in chronically dilated atria. Prog Biophys Mol Biol. (2008) 97:435–51. 10.1016/j.pbiomolbio.2008.02.019 18378284

[6] Staerk L Sherer JA Ko D Benjamin EJ Helm RH. Atrial fibrillation: epidemiology, pathophysiology, and clinical outcomes. Circ Res. (2017) 120:1501–17. 10.1161/CIRCRESAHA.117.309732 28450367 PMC5500874

[7] Freedman B Potpara TS Lip GY. Stroke prevention in atrial fibrillation. Lancet. (2016) 388:806–17. 10.1016/S0140-6736(16)31257-0 27560276

[8] Olesen JB Lip GY Kamper AL Hommel K Kober L Lane DA. Stroke and bleeding in atrial fibrillation with chronic kidney disease. N Engl J Med. (2012) 367:625–35. 10.1056/NEJMoa1105594 22894575

[9] Di Biase L Natale A Romero J. Thrombogenic and arrhythmogenic roles of the left atrial appendage in atrial fibrillation. Circulation. (2018) 138:2036–50. 10.1161/CIRCULATIONAHA.118.034187 30372144

[10] Dukkipati SR Kar S Holmes DR Doshi SK Swarup V Gibson DN. Device-related thrombus after left atrial appendage closure. Circulation. (2018) 138:874–85. 10.1161/CIRCULATIONAHA.118.035090 29752398

[11] Garshick MS Mulliken J Schoenfeld M Riedy K Guo Y Zhong J Average e' velocity on transthoracic echocardiogram is a novel predictor of left atrial appendage sludge or thrombus in patients with atrial fibrillation. Echocardiography. (2018) 35:1939–46. 10.1111/echo.14148 30315597 PMC10723071

[12] Leung DY Black IW Cranney GB Hopkins AP Walsh WF. Prognostic implications of left atrial spontaneous echo contrast in nonvalvular atrial fibrillation. J Am Coll Cardiol. (1994) 24:755–62. 10.1016/0735-1097(94)90025-6 8077549

[13] Yashima N Nasu M Kawazoe K Hiramori K. Serial evaluation of atrial function by Doppler echocardiography after the maze procedure for chronic atrial fibrillation. Euro Heart J. (1997) 18:496–502. 10.1093/oxfordjournals.eurheartj.a015271 9076388

[14] Khan IA. Atrial stunning: basics and clinical considerations. Int J Cardiol. (2003) 92:113–28. 10.1016/S0167-5273(03)00107-4 14659842

[15] Zapolski T Wysokinski A. Stunning of the left atrium after pharmacological cardioversion of atrial fibrillation. Kardiol Pol. (2005) 63:254–62. 16180181

[16] Calkins H Kuck KH Cappato R Brugada J Camm AJ Chen SA. 2012 HRS/EHRA/ECAS expert consensus statement on catheter and surgical ablation of atrial fibrillation: recommendations for patient selection, procedural techniques, patient management and follow-up, definitions, endpoints, and research trial design: a report of the Heart Rhythm Society (HRS) Task Force on Catheter and Surgical Ablation of Atrial Fibrillation. Developed in partnership with the European Heart Rhythm Association (EHRA), a registered branch of the European Society of Cardiology (ESC) and the European Cardiac Arrhythmia Society (ECAS); and in collaboration with the American College of Cardiology (ACC), American Heart Association (AHA), the Asia Pacific Heart Rhythm Society (APHRS), and the Society of Thoracic Surgeons (STS). Endorsed by the governing bodies of the American College of Cardiology Foundation, the American Heart Association, the European Cardiac Arrhythmia Society, the European Heart Rhythm Association, the Society of Thoracic Surgeons, the Asia Pacific Heart Rhythm Society, and the Heart Rhythm Society. Heart Rhythm. (2012) 9:632–96.e21. 10.1093/europace/eum120 22386883

[17] Kottkamp H. Atrial fibrillation substrate: the “unknown species”– from lone atrial fibrillation to fibrotic atrial cardiomyopathy. Heart Rhythm. (2012) 9:481–2. 10.1016/j.hrthm.2012.01.008 22245793

[18] Hirsh BJ Copeland-Halperin RS Halperin JL. Fibrotic atrial cardiomyopathy, atrial fibrillation, and thromboembolism: mechanistic links and clinical inferences. J Am Coll Cardiol. (2015) 65:2239–51. 10.1016/j.jacc.2015.03.557 25998669

[19] Kamel H Healey JS. Cardioembolic Stroke. Circ Res. (2017) 120:514–26. 10.1161/CIRCRESAHA.116.308407 28154101 PMC5312810

[20] Sonaglioni A Vincenti A Baravelli M Rigamonti E Tagliabue E Bassi P. Prognostic value of global left atrial peak strain in patients with acute ischemic stroke and no evidence of atrial fibrillation. Int J Cardiovasc Imaging. (2019) 35:603–13. 10.1007/s10554-018-1485-z 30377893

[21] Nagle RE Smith B Williams DO. Familial atrial cardiomyopathy with heart block. Br Heart J. (1972) 34:205. 5007810

[22] Stables RH Bailey C Ormerod OJ. Idiopathic familial atrial cardiomyopathy with diffuse conduction block. Q J Med. (1989) 71:325–32. 2594963

[23] Jais P Haissaguerre M Shah DC Chouairi S Gencel L Hocini M. A focal source of atrial fibrillation treated by discrete radiofrequency ablation. Circulation. (1997) 95:572–6. 10.1161/01.CIR.95.3.572 9024141

[24] Zipes DP. Electrophysiological remodeling of the heart owing to rate. Circulation. (1997) 95:1745–8. 10.1161/01.CIR.95.7.1745 9107155

[25] Zipes DP. Atrial fibrillation. A tachycardia-induced atrial cardiomyopathy. Circulation. (1997) 95:562–4. 10.1161/01.CIR.95.3.562 9024138

[26] Hoit BD Takeishi Y Cox MJ Gabel M Kirkpatrick D Walsh RA. Remodeling of the left atrium in pacing-induced atrial cardiomyopathy. Mol Cell Biochem. (2002) 238:145–50. 10.1023/A:1019988024077 12349902

[27] Sun H Gaspo R Leblanc N Nattel S. Cellular mechanisms of atrial contractile dysfunction caused by sustained atrial tachycardia. Circulation. (1998) 98:719–27. 10.1161/01.CIR.98.7.719 9715865

[28] Fatkin D Huttner IG Johnson R. Genetics of atrial cardiomyopathy. Curr Opin Cardiol. (2019) 34:275–81. 10.1097/HCO.0000000000000610 30672791

[29] Peng W Li M Li H Tang K Zhuang J Zhang J. Dysfunction of myosin light-chain 4 (MYL4) leads to heritable atrial cardiomyopathy with electrical, contractile, and structural components: evidence from genetically-engineered rats. J Am Heart Assoc. (2017) 6:e007030. 10.1161/JAHA.117.007030 29080865 PMC5721782

[30] Zhong Y Tang K Li H Zhao D Kou W Xu S. Rs4968309 in myosin light chain 4 (MYL4) associated with atrial fibrillation onset and predicts clinical outcomes after catheter ablation in atrial fibrillation patients without structural heart disease. Circ J. (2019) 83:1994–2001. 10.1253/circj.CJ-19-0415 31406021

[31] Disertori M Quintarelli S Grasso M Pilotto A Narula N Favalli V. Autosomal recessive atrial dilated cardiomyopathy with standstill evolution associated with mutation of Natriuretic Peptide Precursor A. Circ Cardiovasc Genet. (2013) 6:27–36. 10.1161/CIRCGENETICS.112.963520 23275345

[32] Tse G Reddy S Chopra J Lee S Liu T Bazoukis G. Electrocardiographic evidence of abnormal atrial phenotype in Brugada syndrome. J Electrocardiol. (2019) 55:102–6. 10.1016/j.jelectrocard.2019.05.005 31152990

[33] Christophersen IE Olesen MS Liang B Andersen MN Larsen AP Nielsen JB. Genetic variation in KCNA5: impact on the atrial-specific potassium current IKur in patients with lone atrial fibrillation. Eur Heart J. (2013) 34:1517–25. 10.1093/eurheartj/ehs442 23264583

[34] Matsubara TJ Fujiu K. Endothelin-1 and atrial cardiomyopathy. Int Heart J. (2019) 60:238–40. 10.1536/ihj.19-039 30890686

[35] Ozcan C Battaglia E Young R Suzuki G. LKB1 knockout mouse develops spontaneous atrial fibrillation and provides mechanistic insights into human disease process. J Am Heart Assoc. (2015) 4:e001733. 10.1161/JAHA.114.001733 25773299 PMC4392447

[36] Bukowska A Lendeckel U Goette A. Atrial calpains: mediators of atrialmyopathies in atrial fibrillation. J Atr Fibrillation. (2014) 6:1021. 10.4022/jafib.1021 27957058 PMC5135235

[37] Goette A Arndt M Rocken C Staack T Bechtloff R Reinhold D. Calpains and cytokines in fibrillating human atria. Am J Physiol Heart Circ Physiol. (2002) 283:H264–72. 10.1152/ajpheart.00505.2001 12063299

[38] Wiedmann F Kiper AK Bedoya M Ratte A Rinne S Kraft M. Identification of the A293 (AVE1231) binding site in the cardiac two-pore-domain potassium channel TASK-1: a common low affinity antiarrhythmic drug binding site. Cell Physiol Biochem. (2019) 52:1223–35. 10.33594/000000083 31001961

[39] Wiedmann F Schulte JS Gomes B Zafeiriou MP Ratte A Rathjens F. Atrial fibrillation and heart failure-associated remodeling of two-pore-domain potassium (K2P) channels in murine disease models: focus on TASK-1. Basic Res Cardiol. (2018) 113:27. 10.1007/s00395-018-0687-9 29881975

[40] Moreau A Janin A Millat G Chevalier P. Cardiac voltage-gated sodium channel mutations associated with left atrial dysfunction and stroke in children. Europace. (2018) 20:1692–8. 10.1093/europace/euy041 29579189

[41] Mazurek T Kiliszek M Kobylecka M Skubisz-Gluchowska J Kochman J Filipiak K. Relation of proinflammatory activity of epicardial adipose tissue to the occurrence of atrial fibrillation. Am J Cardiol. (2014) 113:1505–8. 10.1016/j.amjcard.2014.02.005 24656480

[42] Packer M. Disease-treatment interactions in the management of patients with obesity and diabetes who have atrial fibrillation: the potential mediating influence of epicardial adipose tissue. Cardiovasc Diabetol. (2019) 18:121. 10.1186/s12933-019-0927-9 31551089 PMC6760044

[43] Packer M. Epicardial adipose tissue inflammation can cause the distinctive pattern of cardiovascular disorders seen in psoriasis. Am J Med. (2019). 10.1016/j.amjmed.2019.08.027. [Epub ahead of print]. 31520623

[44] Wang Q Xi W Yin L Wang J Shen H Gao Y. Human epicardial adipose tissue cTGF expression is an independent risk factor for atrial fibrillation and highly associated with atrial fibrosis. Sci Rep. (2018) 8:3585. 10.1038/s41598-018-21911-y 29483593 PMC5827202

[45] Kadappu KK Boyd A Eshoo S Haluska B Yeo AE Marwick TH. Changes in left atrial volume in diabetes mellitus: more than diastolic dysfunction? Eur Heart J Cardiovasc Imaging. (2012) 13:1016–23. 10.1093/ehjci/jes084 22544873

[46] Lin PH Lee SH Su CP Wei YH. Oxidative damage to mitochondrial DNA in atrial muscle of patients with atrial fibrillation. Free Radic Biol Med. (2003) 35:1310–8. 10.1016/j.freeradbiomed.2003.07.002 14607530

[47] Canpolat U. Profibrotic pathways and atrial cardiomyopathy in persistent atrial fibrillation. Clinics. (2016) 71:626. 10.6061/clinics/2016(10)12 27759853 PMC5054772

[48] Kottkamp H Schreiber D The substrate in “early persistent” atrial fibrillation: arrhythmia induced, risk factor induced, or from a specific fibrotic atrial cardiomyopathy? JACC Clin Electrophysiol. (2016) 2:140–2. 10.1016/j.jacep.2016.02.010 29766862

[49] Lin YK Chen YA Lee TI Chen YC Chen SA Chen YJ. Aging modulates the substrate and triggers remodeling in atrial fibrillation. Circ J. (2018) 82:1237–44. 10.1253/circj.CJ-17-0242 28904308

[50] McManus DD Yin X Gladstone R Vittinghoff E Vasan RS Larson MG. Alcohol consumption, left atrial diameter, and atrial fibrillation. J Am Heart Assoc. (2016) 5:e004060. 10.1161/JAHA.116.004060 27628571 PMC5079048

[51] Harada M Van Wagoner DR Nattel S. Role of inflammation in atrial fibrillation pathophysiology and management. Circ J. (2015) 79:495–502. 10.1253/circj.CJ-15-0138 25746525 PMC4457364

[52] Pfluecke C Plichta L Tarnowski D Forkmann M Ulbrich S Quick S. Association of platelet activation markers with recurrence of atrial fibrillation after pulmonary vein isolation. Platelets. (2017) 28:394–9. 10.1080/09537104.2016.1227429 27736274

[53] Tsai LM Chen JH Tsao CJ. Relation of left atrial spontaneous echo contrast with prethrombotic state in atrial fibrillation associated with systemic hypertension, idiopathic dilated cardiomyopathy, or no identifiable cause (lone). Am J Cardiol. (1998) 81:1249–52. 10.1016/S0002-9149(98)00131-3 9604963

[54] Sakurai K Hirai T Nakagawa K Kameyama T Nozawa T Asanoi H. Left atrial appendage function and abnormal hypercoagulability in patients with atrial flutter. Chest. (2003) 124:1670–4. 10.1378/chest.124.5.1670 14605033

[55] Igarashi Y Kashimura K Makiyama Y Sato T Ojima K Aizawa Y. Left atrial appendage dysfunction in chronic nonvalvular atrial fibrillation is significantly associated with an elevated level of brain natriuretic peptide and a prothrombotic state. Jpn Circ J. (2001) 65:788–92. 10.1253/jcj.65.788 11548877

[56] Spronk HM De Jong AM Verheule S De Boer HC Maass AH Lau DH. Hypercoagulability causes atrial fibrosis and promotes atrial fibrillation. Eur Heart J. (2017) 38:38–50. 10.1093/eurheartj/ehw119 27071821

[57] Hasan H Park SH Auger C Belcastro E Matsushita K Marchandot B. Thrombin induces angiotensin II-mediated senescence in atrial endothelial cells: impact on pro-remodeling patterns. J Clin Med. (2019) 8:E1570. 10.3390/jcm8101570 31581517 PMC6833093

[58] Akoum N McGann C Vergara G Badger T Ranjan R Mahnkopf C. Atrial fibrosis quantified using late gadolinium enhancement MRI is associated with sinus node dysfunction requiring pacemaker implant. J Cardiovasc Electrophysiol. (2012) 23:44–50. 10.1111/j.1540-8167.2011.02140.x 21806700 PMC4465539

[59] Akoum N Fernandez G Wilson B McGann C Kholmovski E Marrouche N. Association of atrial fibrosis quantified using LGE-MRI with atrial appendage thrombus and spontaneous contrast on transesophageal echocardiography in patients with atrial fibrillation. J Cardiovasc Electrophysiol. (2013) 24:1104–9. 10.1111/jce.12199 23844972 PMC3818287

[60] Habibi M Lima JA Khurram IM Zimmerman SL Zipunnikov V Fukumoto K. Association of left atrial function and left atrial enhancement in patients with atrial fibrillation: cardiac magnetic resonance study. Circ Cardiovasc Imaging. (2015) 8:e002769. 10.1161/CIRCIMAGING.114.002769 25652181 PMC4319560

[61] Daccarett M Badger TJ Akoum N Burgon NS Mahnkopf C Vergara G. Association of left atrial fibrosis detected by delayed-enhancement magnetic resonance imaging and the risk of stroke in patients with atrial fibrillation. J Am Coll Cardiol. (2011) 57:831–8. 10.1016/j.jacc.2010.09.049 21310320 PMC3124509

[62] Marrouche NF Wilber D Hindricks G Jais P Akoum N Marchlinski F. Association of atrial tissue fibrosis identified by delayed enhancement MRI and atrial fibrillation catheter ablation: the DECAAF study. JAMA. (2014) 311:498–506. 10.1001/jama.2014.3 24496537

[63] Kuppahally SS Akoum N Burgon NS Badger TJ Kholmovski EG Vijayakumar S. Left atrial strain and strain rate in patients with paroxysmal and persistent atrial fibrillation: relationship to left atrial structural remodeling detected by delayed-enhancement MRI. Circ Cardiovasc Imaging. (2010) 3:231–9. 10.1161/CIRCIMAGING.109.865683 20133512

[64] Guo C Liu J Zhao S Teng Y Shen L. Decreased left atrial strain parameters are correlated with prolonged total atrial conduction time in lone atrial fibrillation. Int J Cardiovasc Imaging. (2016) 32:1053–61. 10.1007/s10554-016-0875-3 27076225

[65] Costa C Gonzalez-Alujas T Valente F Aranda C Rodriguez-Palomares J Gutierrez L. Left atrial strain: a new predictor of thrombotic risk and successful electrical cardioversion. Echo Res Pract. (2016) 3:45–52. 10.1530/ERP-16-0009 27249551 PMC4989095

[66] Shih JY Tsai WC Huang YY Liu YW Lin CC Huang YS. Association of decreased left atrial strain and strain rate with stroke in chronic atrial fibrillation. J Am Soc Echocardiogr. (2011) 24:513–9. 10.1016/j.echo.2011.01.016 21353469

[67] Zhu MR Wang M Ma XX Zheng DY Zhang YL. The value of left atrial strain and strain rate in predicting left atrial appendage stasis in patients with nonvalvular atrial fibrillation. Cardiol J. (2018) 25:87–96. 10.5603/CJ.a2017.0069 28612903

[68] Parwani AS Morris DA Blaschke F Huemer M Pieske B Haverkamp W. Left atrial strain predicts recurrence of atrial arrhythmias after catheter ablation of persistent atrial fibrillation. Open Heart. (2017) 4:e000572. 10.1136/openhrt-2016-000572 28674624 PMC5471873

[69] Pathan F Sivaraj E Negishi K Rafiudeen R Pathan S D'Elia N. Use of atrial strain to predict atrial fibrillation after cerebral ischemia. JACC Cardiovasc Imaging. (2018) 11:1557–65. 10.1016/j.jcmg.2017.07.027 29153561

[70] Alonso A Knopman DS Gottesman RF Soliman EZ Shah AJ O'Neal WT. Correlates of dementia and mild cognitive impairment in patients with atrial fibrillation: the atherosclerosis risk in communities neurocognitive study (ARIC-NCS). J Am Heart Assoc. (2017) 6:6014. 10.1161/JAHA.117.006014 28739861 PMC5586306

[71] AlTurki A Maj JB Marafi M Donato F Vescovo G Russo V. The role of cardiovascular and metabolic comorbidities in the link between atrial fibrillation and cognitive impairment: an appraisal of current scientific evidence. Medicina. (2019) 55:767. 10.3390/medicina55120767 31801224 PMC6956022

[72] O'Neal WT Zhang ZM Loehr LR Chen LY Alonso A Soliman EZ. Electrocardiographic advanced interatrial block and atrial fibrillation risk in the general population. Am J Cardiol. (2016) 117:1755–9. 10.1016/j.amjcard.2016.03.013 27072646 PMC4898264

[73] Chen LY Norby FL Gottesman RF Mosley TH Soliman EZ Agarwal SK. Association of atrial fibrillation with cognitive decline and dementia over 20 years: the ARIC-NCS (Atherosclerosis Risk in Communities Neurocognitive Study). J Am Heart Assoc. (2018) 7:7301. 10.1161/JAHA.117.007301 29514809 PMC5907543

[74] Anselmino M Scarsoglio S Saglietto A Gaita F Ridolfi L. Transient cerebral hypoperfusion and hypertensive events during atrial fibrillation: a plausible mechanism for cognitive impairment. Sci Rep. (2016) 6:28635. 10.1038/srep28635 27334559 PMC4917883

[75] Cai Z Liu Z Xiao M Wang C Tian F. Chronic cerebral hypoperfusion promotes amyloid-beta pathogenesis via activating beta/gamma-secretases. Neurochem Res. (2017) 42:3446–55. 10.1007/s11064-017-2391-9 28836062

[76] Bunch TJ Crandall BG Weiss JP May HT Bair TL Osborn JS. Patients treated with catheter ablation for atrial fibrillation have long-term rates of death, stroke, and dementia similar to patients without atrial fibrillation. J Cardiovasc Electrophysiol. (2011) 22:839–45. 10.1111/j.1540-8167.2011.02035.x 21410581

[77] Wu TC. Left atrial stiffness, a marker of atrial cardiomyopathy, and atrial fibrillation - relationships and predictors for procedure success after catheter ablation. Arq Bras Cardiol. (2019) 112:509–10. 10.5935/abc.20190087 31188957 PMC6555573

[78] Blomstrom-Lundqvist C. Atrial fibrillation: from atrial extrasystoles to atrial cardiomyopathy - what have we learned from basic science and interventional procedures? J Intern Med. (2016) 279:406–11. 10.1111/joim.12477 27094102

[79] Pison L Tilz R Jalife J Haissaguerre M. Pulmonary vein triggers, focal sources, rotors and atrial cardiomyopathy: implications for the choice of the most effective ablation therapy. J Intern Med. (2016) 279:449–56. 10.1111/joim.12490 26991806

[80] Disertori M Mase M Marini M Mazzola S Cristoforetti A Del Greco M. Electroanatomic mapping and late gadolinium enhancement MRI in a genetic model of arrhythmogenic atrial cardiomyopathy. J Cardiovasc Electrophysiol. (2014) 25:964–70. 10.1111/jce.12440 24758425

[81] Schreiber D Rieger A Moser F Kottkamp H. Catheter ablation of atrial fibrillation with box isolation of fibrotic areas: lessons on fibrosis distribution and extent, clinical characteristics, and their impact on long-term outcome. J Cardiovasc Electrophysiol. (2017) 28:971–83. 10.1111/jce.13278 28635186

[82] Begg GA Lip GY Plein S Tayebjee MH. Circulating biomarkers of fibrosis and cardioversion of atrial fibrillation: a prospective, controlled cohort study. Clin Biochem. (2017) 50:11–5. 10.1016/j.clinbiochem.2016.09.008 27622867

[83] Markowitz SM Choi DY Daian F Liu CF Cheung JW Thomas G. Regional isolation in the right atrium with disruption of intra-atrial conduction after catheter ablation of atrial tachycardia. J Cardiovasc Electrophysiol. (2019) 30: 1773–85. 10.1111/jce.14037 31225670

[84] Moteleb A Zarif JK Ali AN. Incidence of atrial fibrosis in non-valvular atrial fibrillation patients and its impact on recurrence after pulmonary vein antral isolation. J Atr Fibrillation. (2018) 11:1773. 10.4022/jafib.1773 30455829 PMC6207236

[85] Packer M. Effect of catheter ablation on pre-existing abnormalities of left atrial systolic, diastolic, and neurohormonal functions in patients with chronic heart failure and atrial fibrillation. Eur Heart J. (2019) 40:1873–9. 10.1093/eurheartj/ehz284 31081029 PMC6568203

[86] Greiser M Neuberger HR Harks E El-Armouche A Boknik P de Haan S. Distinct contractile and molecular differences between two goat models of atrial dysfunction: AV block-induced atrial dilatation and atrial fibrillation. J Mol Cell Cardiol. (2009) 46:385–94. 10.1016/j.yjmcc.2008.11.012 19100271

[87] El-Armouche A Boknik P Eschenhagen T Carrier L Knaut M Ravens U. Molecular determinants of altered Ca2+ handling in human chronic atrial fibrillation. Circulation. (2006) 114:670–80. 10.1161/CIRCULATIONAHA.106.636845 16894034

[88] Wakili R Yeh YH Yan Qi X Greiser M Chartier D Nishida K. Multiple potential molecular contributors to atrial hypocontractility caused by atrial tachycardia remodeling in dogs. Circ Arrhythm Electrophysiol. (2010) 3:530–41. 10.1161/CIRCEP.109.933036 20660541

[89] Chiang DY Lebesgue N Beavers DL Alsina KM Damen JM Voigt N. Alterations in the interactome of serine/threonine protein phosphatase type-1 in atrial fibrillation patients. J Am Coll Cardiol. (2015) 65:163–73. 10.1016/j.jacc.2014.10.042 25593058 PMC4690213

[90] Perike S Alsina KM Sridhar A Capote A Martin J Wolska B PPP1R12C regulates myocardial contractility through dephosphorylation of atrial myosin light chain. J Am Coll Cardiol. (2019) 73:827 10.1016/S0735-1097(19)31434-2

[91] Mebazaa A Nieminen MS Packer M Cohen-Solal A Kleber FX Pocock SJ. Levosimendan vs dobutamine for patients with acute decompensated heart failure: the SURVIVE Randomized Trial. JAMA. (2007) 297:1883–91. 10.1001/jama.297.17.1883 17473298

[92] Packer M Colucci W Fisher L Massie BM Teerlink JR Young J. Effect of levosimendan on the short-term clinical course of patients with acutely decompensated heart failure. JACC Heart Fail. (2013) 1:103–11. 10.1016/j.jchf.2012.12.004 24621834

[93] Kivikko M Kuoppamaki M Soinne L Sundberg S Pohjanjousi P Ellmen J Oral levosimendan increases cerebral blood flow velocities in patients with a history of stroke or transient ischemic attack: a pilot safety study. Curr Ther Res Clin Exp. (2015) 77:46–51. 10.1016/j.curtheres.2015.01.001 26082815 PMC4461857

[94] Ellermann C Kohnke A Dechering DG Kochhauser S Reinke F Fehr M. Ranolazine prevents levosimendan-induced atrial fibrillation. Pharmacology. (2018) 102:138-41. 10.1159/000490572 29982246

[95] Anis RR. Role of angiotensin-converting enzyme inhibitors and angiotensin receptor blockers in the management of atrial fibrillation. Exp Clin Cardiol. (2009) 14:e1–7. 19492029 PMC2689089

[96] Li D Shinagawa K Pang L Leung TK Cardin S Wang Z. Effects of angiotensin-converting enzyme inhibition on the development of the atrial fibrillation substrate in dogs with ventricular tachypacing-induced congestive heart failure. Circulation. (2001) 104:2608–14. 10.1161/hc4601.099402 11714658

[97] Shi Y Li D Tardif JC Nattel S. Enalapril effects on atrial remodeling and atrial fibrillation in experimental congestive heart failure. Cardiovasc Res. (2002) 54:456–61. 10.1016/S0008-6363(02)00243-2 12062350

[98] Cohn JN Tognoni G Valsartan Heart Failure Trial I. A randomized trial of the angiotensin-receptor blocker valsartan in chronic heart failure. N Engl J Med. (2001) 345:1667–75. 10.1056/NEJMoa010713 11759645

[99] Investigators S Yusuf S Pitt B Davis CE Hood WB Cohn JN Effect of enalapril on survival in patients with reduced left ventricular ejection fractions and congestive heart failure. N Engl J Med. (1991) 325:293–302. 10.1056/NEJM199108013250501 2057034

[100] Kober L Torp-Pedersen C Carlsen JE Bagger H Eliasen P Lyngborg K. A clinical trial of the angiotensin-converting-enzyme inhibitor trandolapril in patients with left ventricular dysfunction after myocardial infarction. Trandolapril Cardiac Evaluation (TRACE) Study Group. N Engl J Med. (1995) 333:1670–6. 10.1056/NEJM199512213332503 7477219

[101] Anand K Mooss AN Hee TT Mohiuddin SM. Meta-analysis: inhibition of renin-angiotensin system prevents new-onset atrial fibrillation. Am Heart J. (2006) 152:217–22. 10.1016/j.ahj.2006.01.007 16875900

[102] Goette A Schon N Kirchhof P Breithardt G Fetsch T Hausler KG. Angiotensin II-antagonist in paroxysmal atrial fibrillation (ANTIPAF) trial. Circ Arrhythm Electrophysiol. (2012) 5:43–51. 10.1161/CIRCEP.111.965178 22157519

[103] Batra G Lindhagen L Andell P Erlinge D James S Spaak J Angiotensin-converting enzyme inhibitors and angiotensin ii receptor blockers are associated with improved outcome but do not prevent new-onset atrial fibrillation after acute myocardial infarction. J Am Heart Assoc. (2017) 6:5165 10.1161/JAHA.116.005165 PMC5524029 28320744

[104] Miller JD Aronis KN Chrispin J Patil KD Marine JE Martin SS. Obesity, exercise, obstructive sleep apnea, and modifiable atherosclerotic cardiovascular disease risk factors in atrial fibrillation. J Am Coll Cardiol. (2015) 66:2899–906. 10.1016/j.jacc.2015.10.047 26718677

[105] Hatem SN. Atrial fibrillation and obesity: not just a coincidence. J Am Coll Cardiol. (2015) 66:12–3. 10.1016/j.jacc.2015.04.057 26139052

[106] Jamaly S Carlsson L Peltonen M Jacobson P Sjostrom L Karason K. Bariatric surgery and the risk of new-onset atrial fibrillation in swedish obese subjects. J Am Coll Cardiol. (2016) 68:2497–504. 10.1016/j.jacc.2016.09.940 27931605 PMC5157934

[107] Al Chekakie MO Welles CC Metoyer R Ibrahim A Shapira AR Cytron J. Pericardial fat is independently associated with human atrial fibrillation. J Am Coll Cardiol. (2010) 56:784–8. 10.1016/j.jacc.2010.03.071 20797492

[108] Thanassoulis G Massaro JM O'Donnell CJ Hoffmann U Levy D Ellinor PT. Pericardial fat is associated with prevalent atrial fibrillation: the Framingham Heart Study. Circ Arrhythm Electrophysiol. (2010) 3:345–50. 10.1161/CIRCEP.109.912055 20558845 PMC2953855

[109] Wong CX Abed HS Molaee P Nelson AJ Brooks AG Sharma G. Pericardial fat is associated with atrial fibrillation severity and ablation outcome. J Am Coll Cardiol. (2011) 57:1745–51. 10.1016/j.jacc.2010.11.045 21511110

[110] Wong CX Ganesan AN Selvanayagam JB. Epicardial fat and atrial fibrillation: current evidence, potential mechanisms, clinical implications, and future directions. Eur Heart J. (2017) 38:1294–302. 10.1093/eurheartj/ehw045 26935271

